# Supplementary material for: The Evolution and Characterization of the RNA Interference Pathways in Lophotrochozoa
Source: Genome Biol Evol. 2024 May 7;16(5):evae098. doi: 10.1093/gbe/evae098 (PMC11114477; doi:10.1093/gbe/evae098)
Supplement: evae098_Supplementary_Data [file evae098_supplementary_data.zip › Supplementary_materials.zip!/selected_miRNAs/NoGe_selected_miRNAs/mirdeep_pdf_files/NMRB01000004.1_Notospermus_geniculatus_strain_Ushimado_scaffold4,_whole_genome_shotgun_sequence_155.pdf]

| | | |
|---|---|---|
| Provisional ID | : | NMRB01000004.1_Notospermus_geniculatus_strain_Ushimado_scaffold4,_whole_genome_shotgun_sequence_155 |
| Score total | : | 603 |
| Score for star read(s) | : | 3.9 |
| Score for read counts | : | 598.7 |
| Score for mfe | : | 1.1 |
| Score for randfold | : | 0 |
| Score for cons. seed | : | -0.6 |
| Total read count | : | 1186 |
| Mature read count | : | 1171 |
| Loop read count | : | 0 |
| Star read count | : | 15 |

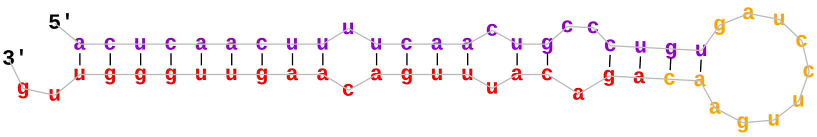


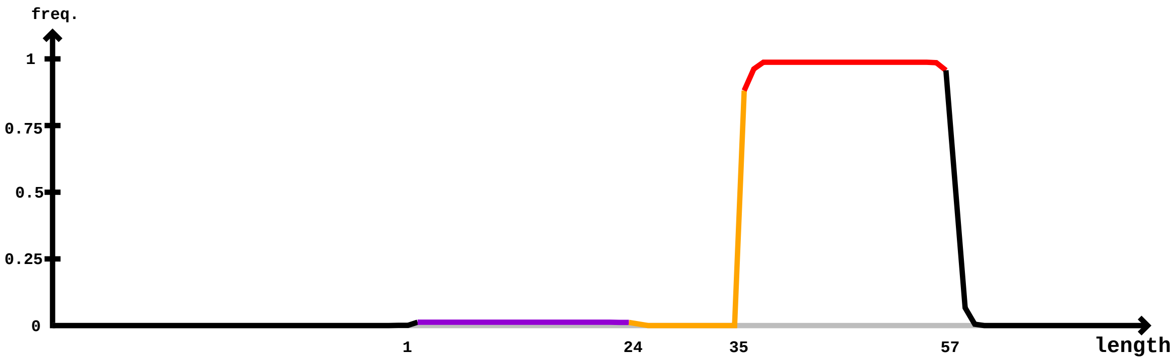


```
                                   Star                              Mature
5'- aacugaagacuaaagugaugggaguugguaauaucgacucaacuuuucaacugcccugugauccuugaacagacauuugacaaguuggguugauguucacaaaccaggcauc   -3'   obs
    aacugaagacuaaagugaugggaguugguaauaucgacucaacuuuucaacugcccugugauccuugaacagacauuugacaaguuggguugauguucacaaaccaggcauc         exp
    ..............(((.(((...(((..((((((((((((((((.((((.((..((((.........)))).)).)))).))))))))))))))))..))).)))..))).   reads     mm     sample
    ....................................acucaacuuuucaacugcccugu.....................................................   1         0      Fm3
    ......................................................................agacauuugacaaguugUguu.....................   1         1      Fm3
    ......................................................................agacauuugacaaguuggguu.....................   10        0      Fm3
    ......................................................................agacauuugacaaguuggguuU....................   1         1      Fm3
    ......................................................................agacauuugacaaguuggguug....................   54        0      Fm3
    ......................................................................agacauuugacaaguuggAuuga...................   1         1      Fm3
    ......................................................................agacauuugacaaguuggguuga...................   18        0      Fm3
    ......................................................................agacauuugacaaguuggguugU...................   41        1      Fm3
    ......................................................................agacauuugacaaguuggguugau..................   2         0      Fm3
    .......................................................................gacauuugacaaguuggguug....................   2         0      Fm3
    .......................................................................gacauuugacaaguuggguugU...................   2         1      Fm3
    .......................................................................gacauuugacaaguuggguuga...................   14        0      Fm3
    .......................................................................gacauuugacaaguuggguugaA..................   6         1      Fm3
    .......................................................................gacauuugacaaguuggguugau..................   3         0      Fm3

    ....................................acucaacuuuucaacugcccugu.....................................................   4         0      Mm3
    ......................................................................agacauuugacaaguuggguu.....................   2         0      Mm3
    ......................................................................agacauuugacaaguuggguuC....................   1         1      Mm3
    ......................................................................agacauuugacaaguuggguug....................   10        0      Mm3
    ......................................................................agacauuugacaaguuggguuU....................   1         1      Mm3
    ......................................................................agacauuugacaaguuggguugU...................   45        1      Mm3
    ......................................................................agacauuugacaaguuggguuga...................   3         0      Mm3
    ......................................................................agacauuugacaaguuggguugaA..................   1         1      Mm3
    ......................................................................agacauuugacaaguuggguugUu..................   3         1      Mm3
    ......................................................................agacauuugacaaguuggguugau..................   2         0      Mm3

    ......................................................................agacauuugacaaguuggguu.....................   14        0      Fm2
    ......................................................................agacauuugacaaUuuggguu.....................   1         1      Fm2
    ......................................................................agacauuugacaaguuggguug....................   180       0      Fm2
    ......................................................................agacauuugacaaguuggguGg....................   1         1      Fm2
    ......................................................................agacauuugacaaguugCguug....................   3         1      Fm2
    ......................................................................agacauuugacaGguuggguug....................   4         1      Fm2
    ......................................................................agacauuugacaaguugAguug....................   2         1      Fm2
```

Star Mature

| | | | |
|---|---|---|---|
| aacugaagacuaaagugaugggaguugguaauaucgacucaacuuuucaacugcccugugauccuugaacagacauuugacaaguuggguugauguucacaaaccaggcauc | | | |
| ......................................................................agacauuugacaaguuggguugU................... | 94 | 1 | Fm2 |
| ......................................................................agacauuugacaGguuggguuga................... | 4 | 1 | Fm2 |
| ......................................................................agacGuuugacaaguuggguuga................... | 1 | 1 | Fm2 |
| ......................................................................agacauuugacaaguuggguuga................... | 50 | 0 | Fm2 |
| ......................................................................agacauuugacaaguuggguugG................... | 2 | 1 | Fm2 |
| ......................................................................agacauuugacaaguuggguugau.................. | 5 | 0 | Fm2 |
| ......................................................................agacauuugacaaguuggguugUu.................. | 8 | 1 | Fm2 |
| .......................................................................gacauuugacaaguuggguug.................... | 6 | 0 | Fm2 |
| .......................................................................gacauuugacaaguuggguuga................... | 41 | 0 | Fm2 |
| .......................................................................gacauuugacaaguuggAuuga................... | 1 | 1 | Fm2 |
| ........................................................................acauuugacaaguuggguugau.................. | 9 | 0 | Fm2 |
| ........................................................................acauuugacaaguuggguugaA.................. | 2 | 1 | Fm2 |
| ......................................................................agacauuugacaaguuggguCg.................... | 1 | 1 | Fm1 |
| ......................................................................agacauuugacaaguuCgguug.................... | 1 | 1 | Fm1 |
| ......................................................................agacauuugacaaguugCguug.................... | 1 | 1 | Fm1 |
| ......................................................................aAacauuugacaaguuggguug.................... | 1 | 1 | Fm1 |
| ......................................................................agacauuugacaaguuggguug.................... | 62 | 0 | Fm1 |
| ......................................................................agacauuugacaaguuggguGg.................... | 2 | 1 | Fm1 |
| ......................................................................agacauuugacaaguuggguuga................... | 11 | 0 | Fm1 |
| ......................................................................agacauuugacaaguuggguugU................... | 70 | 1 | Fm1 |
| ......................................................................agacauuugacaaguuggguugG................... | 1 | 1 | Fm1 |
| ......................................................................agacauuugacaaguuggguugaA.................. | 2 | 1 | Fm1 |
| ......................................................................agacauuugacaaguuggguugau.................. | 3 | 0 | Fm1 |
| ......................................................................agacauuugacaaguuggguugUu.................. | 4 | 1 | Fm1 |
| .......................................................................gacauuugacaaguuggguuga................... | 8 | 0 | Fm1 |
| ........................................................................acauuugacaaguugggCugau.................. | 1 | 1 | Fm1 |
| ........................................................................acauuugacaaguuggguugau.................. | 4 | 0 | Fm1 |
| ........................................................................acauuugacaaguuggguugauA................. | 5 | 1 | Fm1 |
| ......................................................................agacauuugacaaguugggu...................... | 1 | 0 | Mm1 |
| ......................................................................agacauuugacaaguuggguug.................... | 14 | 0 | Mm1 |
| ......................................................................agacauuugacaaguuggguugU................... | 24 | 1 | Mm1 |
| ......................................................................agacauuugacaaguuggguugUu.................. | 3 | 1 | Mm1 |
| ..................................cgacucaacuuuucaacugcccG....................................................... | 1 | 1 | Mm2 |
| ....................................acucaacuuuucaacugcccugu..................................................... | 9 | 0 | Mm2 |
| ......................................................................agacauuugacaaguugggu...................... | 1 | 0 | Mm2 |
| ......................................................................agacauuugacaaguuggguu..................... | 6 | 0 | Mm2 |
| ......................................................................agacauuugacaaguuggguuU.................... | 1 | 1 | Mm2 |
| ......................................................................agacauuugacaaguuUgguug.................... | 1 | 1 | Mm2 |
| ......................................................................agacauuugacaaguugAguug.................... | 1 | 1 | Mm2 |
| ......................................................................agacauuugCcaaguuggguug.................... | 1 | 1 | Mm2 |
| ......................................................................agacauuugacaaguuggguug.................... | 62 | 0 | Mm2 |
| ......................................................................agacauuugaUaaguuggguug.................... | 2 | 1 | Mm2 |
| ......................................................................agacauuugacaaguCggguug.................... | 1 | 1 | Mm2 |
| ......................................................................agacauuugacaaguuggguugG................... | 2 | 1 | Mm2 |
| ......................................................................agacauuugacaaguuggguugU................... | 170 | 1 | Mm2 |
| ......................................................................agacauuugacaaguuggguuga................... | 22 | 0 | Mm2 |
| ......................................................................agacauuugacaaguuggguugUu.................. | 6 | 1 | Mm2 |
| ......................................................................agacauuugacaaguuggguugau.................. | 2 | 0 | Mm2 |
| ......................................................................agacauuugacaaguuggguugaG.................. | 1 | 1 | Mm2 |
| .......................................................................gacauuugacaaguuggguuga................... | 13 | 0 | Mm2 |
| ........................................................................acauuugacaaguuggguuga................... | 1 | 0 | Mm2 |
| ........................................................................acauuugacaaguuggguugU................... | 1 | 1 | Mm2 |
| ........................................................................acauuugacaaguuggguugau.................. | 6 | 0 | Mm2 |
| ........................................................................acauuugacaaguuggguugauU................. | 1 | 1 | Mm2 |
